# Systolic reverse flow derived from 4D flow cardiovascular magnetic resonance in bicuspid aortic valve is associated with aortic dilation and aortic valve stenosis: a cross sectional study in 655 subjects

**DOI:** 10.1186/s12968-022-00906-9

**Published:** 2023-01-26

**Authors:** Elizabeth K. Weiss, Kelly Jarvis, Anthony Maroun, S. Chris Malaisrie, Christopher K. Mehta, Patrick M. McCarthy, Robert O. Bonow, Ryan J. Avery, Bradley D. Allen, James C. Carr, Cynthia K. Rigsby, Michael Markl

**Affiliations:** 1 Department of Radiology, Feinberg School of Medicine, Northwestern University, 737 North Michigan Avenue Suite 1600, Chicago, IL 60611 USA; 2 Division of Cardiac Surgery, Feinberg School of Medicine, Northwestern University, Chicago, IL USA; 3 Division of Cardiology, Feinberg School of Medicine, Northwestern University, Chicago, IL USA; 4 Department of Medical Imaging, Lurie Children’s Hospital, Chicago, IL USA

**Keywords:** Bicuspid aortic valve, Aortic dilation, Aortic stenosis, Aortic regurgitation, 4D flow MRI, Voxel-wise reverse flow

## Abstract

**Background:**

Bicuspid aortic valve (BAV) disease is associated with increased risk of aortopathy. In addition to current intervention guidelines, BAV mediated changes in aortic 3D hemodynamics have been considered as risk stratification measures. We aimed to evaluate the association of 4D flow cardiovascular magnetic resonance (CMR) derived voxel-wise aortic reverse flow with aortic dilation and to investigate the role of aortic valve regurgitation (AR) and stenosis (AS) on reverse flow in systole and diastole.

**Methods:**

510 patients with BAV (52 ± 14 years) and 120 patients with trileaflet aortic valve (TAV) (61 ± 11 years) and mid-ascending aorta diameter (MAAD) > 35 mm who underwent CMR including 4D flow CMR were retrospectively included. An age and sex-matched healthy control cohort (n = 25, 49 ± 12 years) was selected. Voxel-wise reverse flow was calculated in the aorta and quantified by the mean reverse flow in the ascending aorta (AAo) during systole and diastole.

**Results:**

BAV patients without AS and AR demonstrated significantly increased systolic and diastolic reverse flow (222% and 13% increases respectively, p < 0.01) compared to healthy controls and also had significantly increased systolic reverse flow compared to TAV patients with aortic dilation (79% increase, p < 0.01). In patients with isolated AR, systolic and diastolic AAo reverse flow increased significantly with AR severity (c = − 83.2 and c = − 205.6, p < 0.001). In patients with isolated AS, AS severity was associated with an increase in both systolic (c = − 253.1, p < 0.001) and diastolic (c = − 87.0, p = 0.02) AAo reverse flow. Right and left/right and non-coronary fusion phenotype showed elevated systolic reverse flow (> 17% increase, p < 0.01). Right and non-coronary fusion phenotype showed decreased diastolic reverse flow (> 27% decrease, p < 0.01). MAAD was an independent predictor of systolic (p < 0.001), but not diastolic, reverse flow (p > 0.1).

**Conclusion:**

4D flow CMR derived reverse flow associated with BAV was successfully captured even in the absence of AR or AS and in comparison to TAV patients with aortic dilation. Diastolic AAo reverse flow increased with AR severity while AS severity strongly correlated with increased systolic reverse flow in the AAo. Additionally, increasing MAAD was independently associated with increasing systolic AAo reverse flow. Thus, systolic AAo reverse flow may be a valuable metric for evaluating disease severity in future longitudinal outcome studies.

## Introduction

Bicuspid aortic valve (BAV) disease is the most common congenital heart defect, with a prevalence of 1–2% [1] in the general population. While BAV is often asymptomatic and left undiagnosed in childhood, [2] BAV poses significant cardiovascular risk in adults. In particular, BAV disease increases the risk of the development of aortopathy such as aortic dilation, aneurysm, and dissection [3–6]. To prevent complications, surgical aortic replacement is considered the primary preventative strategy. However, current guidelines for the timing of BAV surgery are based on simple metrics and have undergone several changes in the last decades [7].

The most recent guidelines recommend aortic dimension as the primary indicator for surgery [8]. However, a large collection of additional risk factors, such as aortic growth rate > 5 mm/year, uncontrolled hypertension, and dilation phenotype, can influence the decision to refer the patient for surgery. In addition, severe aortic stenosis (AS) and aortic regurgitation (AR) are an indication for valve replacement and often result in surgical aortic replacement in less dilated aortas. These multitude of risk factors ultimately leads to non-standardized initiation of surgical repair.

As 4D flow cardiovascular magnetic resonance (CMR) enters clinical use, BAV mediated changes in aortic 3D hemodynamics have been considered as new risk stratification measures [9, 10]. It is well established that bicuspid aortic valve patients present with significantly altered aortic 3D flow dynamics compared to subjects with normal tricuspid aortic valves, even in the absence of AS or AR [11, 12]. 4D flow CMR studies have shown that the altered BAV morphology can result in aberrant aortic flow including elevated pulse wave velocity [13], increased wall shear stress (WSS) [14, 15], and presence of marked vortical and helical flow in the ascending aorta (AAo) [16].These metrics capture BAV mediated changes beyond what can be extracted from anatomical imaging and are being explored as potential risk factors for BAV aortopathy. Increased vortex flow and WSS, for example, are associated with larger aortic dimensions and aortic wall degeneration on histopathology [17, 18] and more rapid progression of aortic dilation [10]. It has also been demonstrated that AS and AR can exacerbate these effects and result in further elevated peak velocities and ascending aortic wall shear stress (WSS) [19, 20].

However, the quantification of advanced hemodynamic metrics, such as WSS, has several limitations that hinder its wider application. These include non-standardized and complex techniques, noise and error propagation, and sensitivity to aorta wall motion, segmentation and spatial resolution [16, 21, 22]. A prior study found that 4D flow derived reverse flow, a direct, simple measure, was increased in BAV patients and that those patients requiring surgical intervention had significantly increased reverse flow compared to those not requiring intervention [23]. However, the analysis required substantial manual interaction. We propose a semi-automated method to measure 4D flow CMR derived voxel-wise aortic reverse flow to quantify flow derangement in BAV patients. Our study aimed to investigate the role of aortic valve morphology, aortic dilation, as well as AS and AR severity in driving reverse flow in systole and diastole in a large, retrospective cross-sectional study in BAV patients and controls groups with normal trileaflet aortic valves (TAV).

The goal of this study was to test the hypothesis that reverse flow captures bicuspid aortic valve driven changes in hemodynamics in systole and diastole. We further aimed to assess the independent contributions of AR and AS to systolic and diastolic reverse flow in BAV patients. We lastly aimed to investigate the relationship between reverse flow and degree of aortic dilation in BAV patients.

## Methods

### Study cohort

This retrospective study utilized an institutional database consisting of 1178 adult patients with bicuspid aortic valve who underwent standard-of-care CMR for surveillance of aortic dilation and/or aortic valve disease between November 1, 2011 and September 30, 2020. Subjects were included if they had aortic dilatation, defined by mid-ascending aortic diameter (MAAD) > 35 mm (726 patients included), as measured by contrast enhance CMR angiography. This threshold was based on previously reported upper normal limits of ascending aorta diameter [24]. Exclusion criteria included a history of aorta or aortic valve surgery (46 excluded) and a clinical history of connective tissue disorder (1 patient excluded). Further, only those with a 4D flow data set available for preprocessing by an automated pipeline were analyzed (162 excluded). These criteria yielded 535 BAV patients suitable for inclusion. AR and AS status were determined from radiologist impression on CMR report, using 2D phase-contrast CMR and quantitative grading guidelines [8, 25], and sorted into 4 groups: none, mild, moderate, severe. Those with intermediate determinations (i.e., mild-moderate) were grouped into the higher severity group. A database of 824 patients with TAV was utilized to assemble a TAV control cohort with aortic dilatation. Patients were included if they had an MAAD > 35 mm and preprocessed by an automated pipeline. Patients were excluded if they had AR or AS or history of cardiac surgery. These criteria yielded a cohort of 120 TAV subjects. Cardiac function metrics for all subjects were measured by short-axis CINE and collected from radiology report. Additionally, a healthy control cohort (n = 25) was selected from a separate database of heathy subjects who underwent an IRB approved research 4D flow CMR exam. Individuals were selected to match the age and sex distribution to the bicuspid aortic valve cohort. These subjects did not undergo contrast-enhanced CMR angiography and thus did not have aortic dimensions measured. This HIPAA-compliant study was approved by the Institutional Review Board (IRB). Patients were retrospectively enrolled with a waiver of consent, while controls provided written informed consent per IRB requirement.

### CMR imaging

All CMR exams were performed on 1.5 T or 3 T CMR systems (Avanto, Aera, and Skyra, Siemens Healthineers, Erlangen, Germany). Each patient underwent standard-of-care CMR, including a gadolinium enhanced CMR angiogram, followed by 4D flow CMR with complete coverage of the thoracic aorta (respiratory navigator gated, sagittal oblique, with either retrospective or prospective cardiac gating). 4D flow CMR scan parameters were as follows: spatial resolution = 2.38 × 1.77 × 2.4 mm^3^ − 3.88 × 2.63 × 4 mm^3^, FOV = 340–470 × 234–385 mm, TR = 36–42 ms, TE = 2.03–2.8 ms, flip angle = 7–15° and velocity encode (venc) = 150–300 cm/s.

### 4D flow CMR data analysis—preprocessing and 3D segmentation

Each scan was automatically preprocessed for phase offset errors (Maxwell terms, eddy currents), noise-masking, and velocity antialiasing [26] using an in-house deep-learning pipeline. Eddy current correction used an automatically determined static tissue mask in conjunction with previously established correction methods [27]. A previously described deep-learning method [28] was employed for fully automated 3D segmentation of the thoracic aorta for all subjects. Supra-aortic branches were excluded from the 3D segmentation using a manually drawn region of exclusion. The segmentation was used to mask the 4D flow velocity data and was interpolated to 1 mm isotropic resolution.

**Fig. 1:**
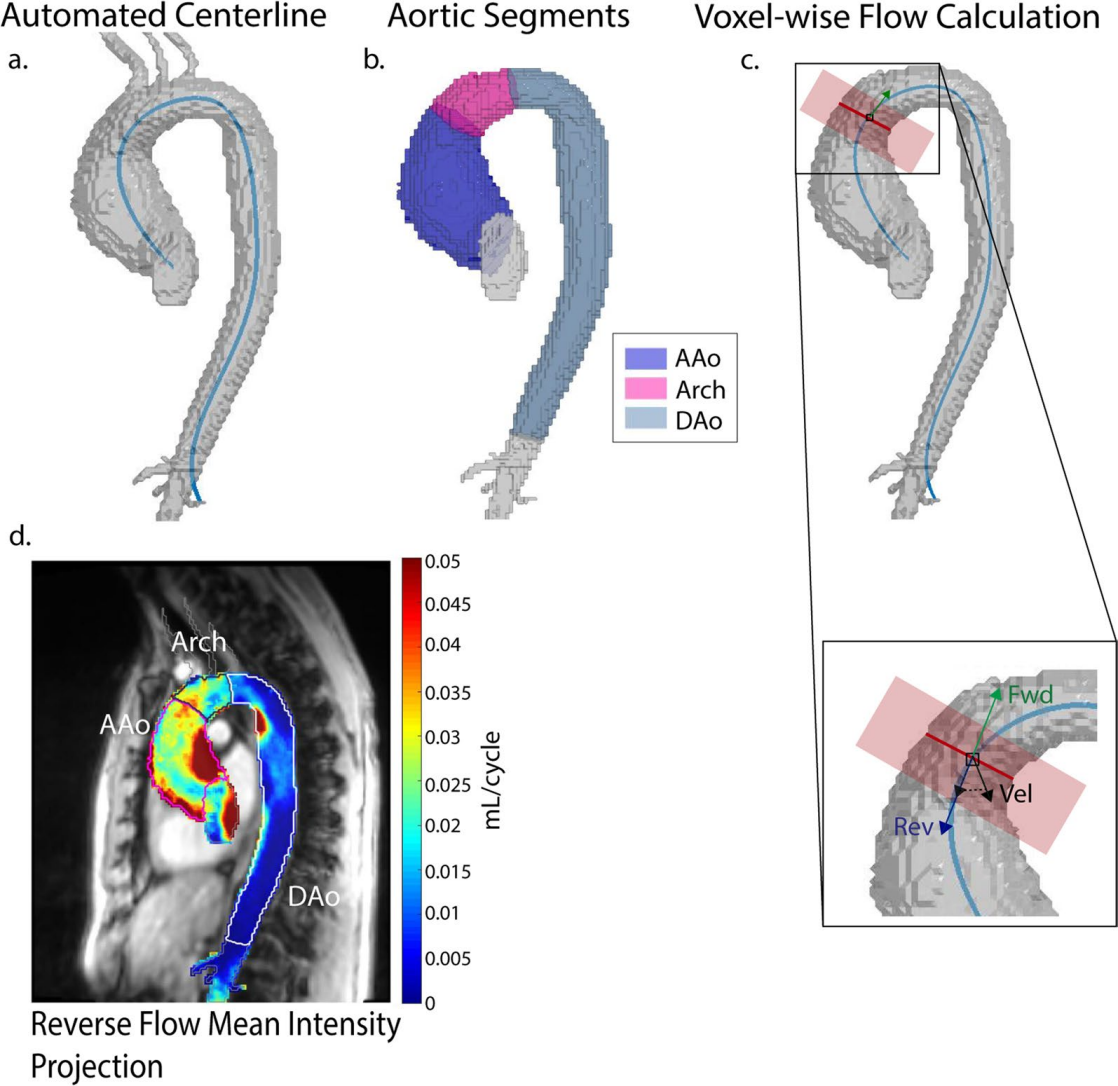
Analysis workflow. Following AI segmentation of the aorta, an automated centerline was calculated (**a**). Analysis planes were placed to delineate 3 aortic segments (AAo, aortic arch, and DAo) and supra-aortic branches were removed (**b**). Analysis planes were automatically placed every millimeter along the centerline to determine the direction of forward flow (green) and reverse flow (blue). The velocity at each voxel (Vel) associated with this plane was projected onto the reverse flow vector (Rev) to calculate the reverse flow (**c**). 2D mean intensity projections of reverse flow were generated (**d**) and mean voxel-wise reverse flow was calculated for each aortic segment

Based on the 3D aorta segmentation, an automated aorta centerline was calculated, and 2D analysis planes were manually placed at the sinotubular junction, proximal to the first supra-aortic branch, distal to the left subclavian artery, and in the descending aorta proximal to the celiac artery. The four 2D planes delineate three aortic segments (Fig. 1a, b): AAo, aortic arch, and descending aorta (DAo). Planes were placed such that the left ventricular outflow tract and DAo beyond the celiac artery were excluded from the analysis.

### 4D flow CMR data analysis—reverse flow maps

2D orthogonal analysis planes were automatically placed every 1 mm along the entire aortic centerline. At each plane, the unit normal vector, defining forward (away from the aortic valve) and reverse flow (towards the aortic valve), was calculated. Each voxel within the 3D aorta segmentation was assigned to the nearest 2D analysis plane to define the direction of flow at every voxel (Fig. 1c). The velocities were projected onto the forward and reverse unit normal vectors to determine the respective voxel-wise contribution to forward and reverse flow. This method was adapted from previous work [29, 30].

Voxel-wise forward and reverse flow was calculated for all cardiac time points. To account for inter-individual differences in heart rate and electrocardiogram gating (495 prospective, 18 retrospective), only cardiac time frames covering the shortest RR-interval acquired in all subjects (600 ms, a prospectively gated acquisition) were used for flow analysis. The number of cardiac timepoints included varied from 14 to 17, depending on the temporal resolution. End systole was individually determined for each patient and healthy control using the mean net flow over the entire aortic segmentation. The first inflection point following peak systole was selected as end systole. For each voxel, total reverse flow was then calculated for systole, diastole and the whole cardiac cycle (Fig. 1d). These measures were termed systolic reverse flow, diastolic reverse flow, and whole cycle reverse flow, respectively. Mean reverse flow (average over all voxels) was calculated for each of the three aortic segments (AAo, arch, DAo). The reported measures reflect the average reverse flow for a single 1 mm^3^ voxel. Thus, all measurements were expected to be small in magnitude compared to typical clinical measures of reverse flow. To visualize regional flow reversal, all 3D flow maps were averaged along the sagittal direction to generate 2D mean intensity projection reverse maps (Fig. 1d). To provide clinical context for the small, voxel-wise values measured, an estimate of total reverse flow through a 2D ROI at the mid-ascending aorta was calculated for 2 example patients with bicuspid aortic valve. The average MAAD for the BAV cohort (42.8 mm) was used. It was assumed that each voxel in the plane experienced the measured, mean systolic reverse flow in the ascending aorta.

To investigate inter-observer variability due to the manual interaction required by the analysis pipeline, measurement of ascending aortic systolic and diastolic reverse flow was repeated for 30 randomly selected BAV patient scans by a second observer. Bias and limits of agreement (LOA) were determined by Bland–Altman analysis.

### Statistical analysis

All data are reported as mean ± SD. One-way ANOVA was used, followed by pairwise t-tests, to assess for differences in age, sex, heart rate, cardiac output, stroke volume (SV), left ventricular (LV) end systolic volume (LVESV), and end diastolic volume (LVEDV) across AR stratifications and the healthy control cohort.

Systolic, diastolic and whole cycle reverse flow in each aortic segment and reverse flow volume fraction were compared across BAV patient groups with isolated AS or AR and compared to the healthy controls with Bonferroni corrected rank-sum tests. In those with pure AR, logistic regression was used to assess the correlative relationships between reverse flow and AR severity. This was similarly repeated in those with pure AS. The non-intercept terms (reported as c) were reported. To investigate the interactions between AS and AR severity on reverse flow, one-way ANOVA was used.

BAV subjects with valve morphology indicated at time of database query were included and had no-mild AR and AS were included in a sub-group analysis (n = 170). These subjects were stratified by valve morphology. For morphologies with more than 10 subjects, systolic and diastolic reverse flow in the AAo were compared using Bonferroni corrected Wilcoxon rank-sum tests.

Multiple linear regression was used to assess independent relationships between reverse flow, valve disease severity, aortic diameter, cardiac function, and subject characteristics. AS, AR, SV, LV ejection fraction (LVEF), age, gender and MAAD were included as possible independent predictors of reverse flow. Linear regression was preformed twice with different dependent variables: (1) mean systolic reverse flow in the AAo and (2) mean diastolic reverse flow in the AAo. All subjects in the BAV cohort were included in the analysis.

## Results

### Study cohort

Of the 535 patients who fulfilled initial inclusion and exclusion criteria, 17 were excluded due to high levels of noise in 4D flow CMR acquisition and inability to segment the aorta and 3 were excluded due to lack of radiology report in the electronic medical record. In addition, 5 data sets were excluded due to inability to automatically calculate an appropriate aorta center line. A total of 510 BAV subjects, 120 TAV subjects, and 25 healthy control subjects were included in the final study cohort.

The demographics of the study cohort are summarized in Table 1. There were no significant differences in age, body mass index (BMI), heart rate and cardiac output between the healthy controls and any AR subgroup (no, mild, moderate, or severe AR). The TAV with dilation cohort was significantly older than the no AR BAV subgroup (61.1 vs. 52.5, p < 0.001). There were 128 patients without aortic regurgitation or stenosis, 180 with isolated AR, 88 with isolated AS and 122 with both AR and AS. The BAV subgroup without AR had a significantly larger proportion of females compared to all other subgroups, but no subgroup was significantly different from the control cohort. Compared to healthy and TAV with dilation controls, BAV patients without AR had higher LVEF (56.1% and 59.1% vs. 62.0%, p < 0.001). Compared to healthy controls, BAV subjects without AR had reduced LVESV (67 mL vs. 56.6 mL, p < 0.001). BAV patients with mild AR presented with higher LVEF (56.1% vs. 60.5%, p < 0.001) and SV (84.5 mL vs. 101 mL, p < 0.0025) compared to controls. In addition, ESV was elevated in the severe AR group (67 mL vs. 124 mL, p < 0.001) while LVEDV and LV SV were increased in both the moderate (152 mL vs. 194 mL, p < 0.001 and 84.5 mL vs. 117 mL, p < 0.001) and severe AR groups (152 mL vs. 261 mL, p < 0.001 and 84.5 mL vs. 140 mL, p < 0.001) compared to healthy controls. SV, LVESV, and LVEDV were significantly different between every pairwise comparison of the BAV subgroups. EF was significantly lower in those with severe AR compared to those with no or mild AR (55.2% vs. 62.7% and 60.5%, p < 0.001). There was a significantly smaller proportion of subjects with concurrent severe AS in the moderate AR subgroup compared to the no AR and severe AR subgroups (5.7% vs. 19.4% and 25% p < 0.001). Among BAV subjects there were no significant differences in MAAD across the four AR groups or across the four AS groups. There was also no significant difference in MAAD between BAV subjects without AR or AS and those with TAV and dilation. However, the sinus of valsalva diameter was significantly larger in the TAV patients with dilation compared to BAV patients without AR (42.1 mm vs. 40.0 mm, p < 0.001).

**Table 1:** Demographics for the healthy control and BAV cohorts

	Healthy control	TAV + dilation	All BAV	BAV − No AR	BAV − mild AR	BAV − moderate AR	BAV − severe AR	p-value
Group size	25	120	510	216	171	87	36	–
Age (years)	49 ± 12	61.1 ± 10.7^**□**^	51.5 ± 13.5	52.5 ± 14	52.2 ± 12.6	48.8 ± 13.7	48.3 ± 12	**< 0.001**
BMI	26 ± 5	28.9 ± 5.7	27.9 ± 5.6	28.1 ± 6.4	27.9 ± 5.2	27.5 ± 4.3	28.3 ± 4.1	0.4
Female (%)	24.0	21.7	24.4	35.1^∆○◊^	20.7^□^	11.5^□^	8.3^□^	**< 0.001**
LVEF (%)	56.1 ± 6.2	59.1 ± 5.8^**□**^	60.7 ± 7.9	62 ± 7.2^*◊^	60.5 ± 7.7^*◊^	59.8 ± 8.7	55.2 ± 9	**< 0.001**
MAAD (mm)	–	40.7 ± 3.5	42.8 ± 5	42.2 ± 4.9	43.5 ± 5.2	42.9 ± 4.6	43.2 ± 4.8	**< 0.001**
SOV (mm)	–	42.1 ± 5.1^**□**^	41.1 ± 4.9	40 ± 5	41.7 ± 4.7	42 ± 4.9	42.6 ± 4.2	**< 0.001**
HR	64 ± 9	64 ± 11	69 ± 12	68 ± 12	69 ± 12	69 ± 13	69 ± 2	0.5
CO	5.3 ± 1.1	5.3 ± 1.5^**□**^	7.8 ± 12.4	7.6 ± 15.3	6.5 ± 1.9	10.4 ± 18	9.4 ± 2.4	**0.04**
SV	84.5 ± 16.7	84.4 ± 25.5	101 ± 33	88.7 ± 27^∆○◊^	101 ± 30^□○◊^	117 ± 30^*□∆◊^	140 ± 34^*□∆○^	**< 0.001**
LVESV	67 ± 17.7	59.2 ± 20.9	69.3 ± 37	56.6 ± 27^*∆○◊^	68.9 ± 33^□○◊^	79.6 ± 32.3^□∆◊^	124 ± 57^*□∆○^	**< 0.001**
LVEDV	152 ± 29	144 ± 43	170 ± 61	146 ± 47^∆○◊^	170 ± 53^□○◊^	194 ± 52^*□∆◊^	261 ± 79^*□∆○^	**< 0.001**
No AS	–	120	308	128	106	55	19	0.7
Mild AS	–	–	55	19	23	11	2	0.4
Moderate AS	–	–	68	27	19	16	6	0.4
Severe AS	–	–	79	42^○^	23	5^◊□^	9^○^	**0.007**

Significant difference compared to *—Healthy Control, □—No AR, ∆—Mild AR, ○—Moderate AR, ◊—Severe AR

One way ANOVA compared healthy controls, TAV patients with dilation, and AR subgroups (p-value reported). The legend indicates which group a value is significantly different (p < 0.005) from. TAV + dilation was only compared with BAV − no AR group. Blood pressure was not available in most subjects and was excluded from this table. AR, aortic regurgitation; BAV, bicuspid aortic valve; CO, cardial output; LVEDV, left ventricular end-diastolic volume, LVEF, left ventricular ejection fraction; LVESV, left ventricular end-systolic volume, MAAD, mid ascending aorta diameter; SV, stroke volume; TAV, trileaflet aortic valve

### Reverse flow maps and interobserver repeatability

**Fig. 2:**
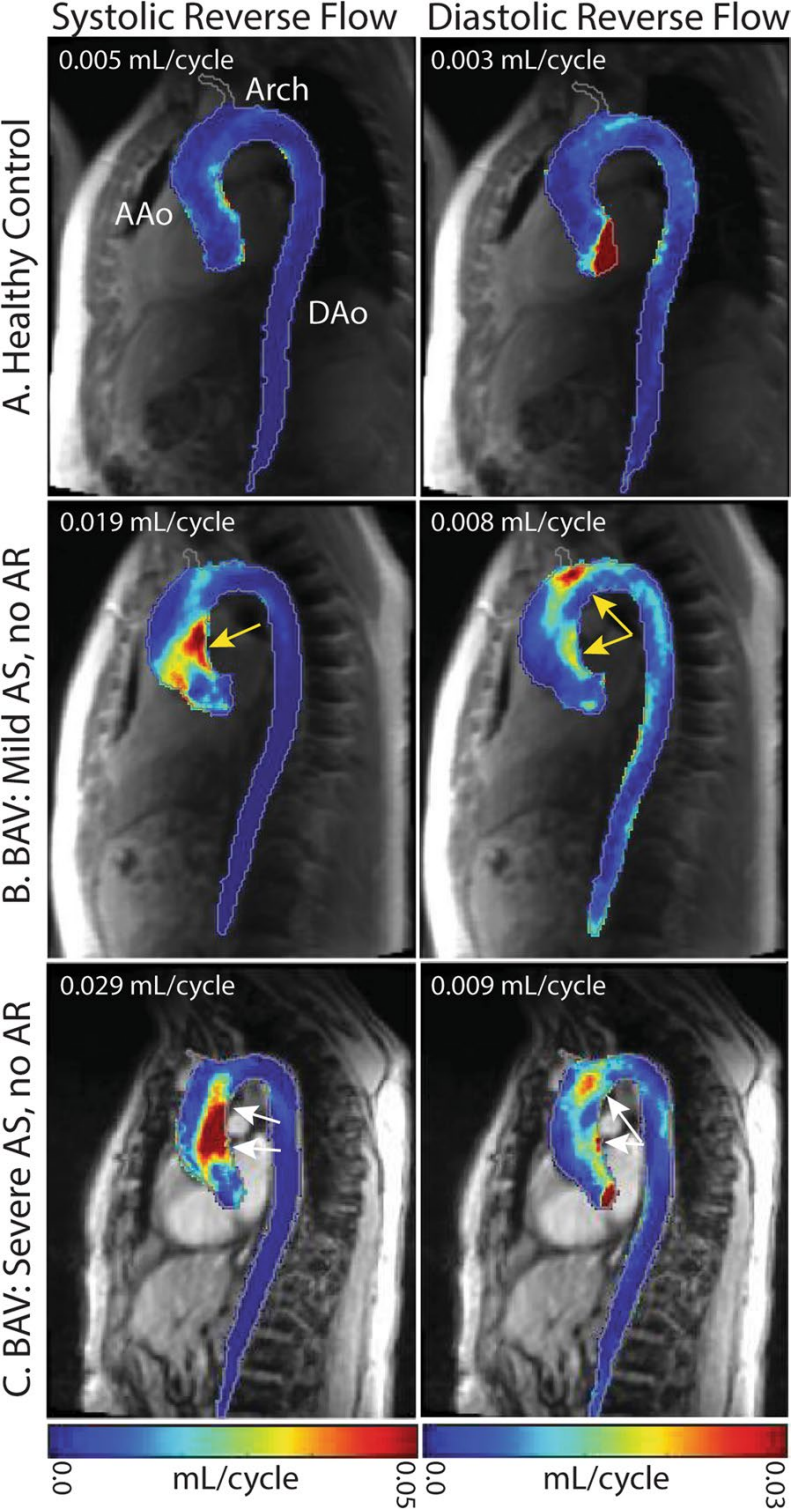
Reverse flow mean intensity projections of the aorta in a healthy control and BAV patients with isolated AS. Voxel-wise reverse flow in the AAo is reported in the upper left corner of each image. Systolic and diastolic reverse flow were increased in a subject with mild AS in the AAo (**b**, yellow arrows) compared to a healthy control (**a**). Systolic reverse flow was further increased in a subject with severe AS (**c**, white arrows)

Figure 2 illustrates representative examples of reverse flow maps in BAV patients with isolated AS and no AR compared to a healthy control subject. Despite the absence of AR, reverse flow in the AAo and arch was increased in the BAV patients compared to the healthy control subject. Even mild AS in the absence of AR was associated with increased reverse flow in the AAo and aortic arch during both systole and diastole compared to a healthy control subject. For the BAV patient with severe isolated AS (no AR) a marked increase in systolic reverse flow in the AAo compared to mild AS (0.029 vs. 0.019 mL/cycle) was evident. In contrast, diastolic reverse flow in the AAo and aortic arch was similar for mild and severe AS, demonstrating that increased AS severity predominantly impacted systolic AAo flow patterns.

**Fig. 3:**
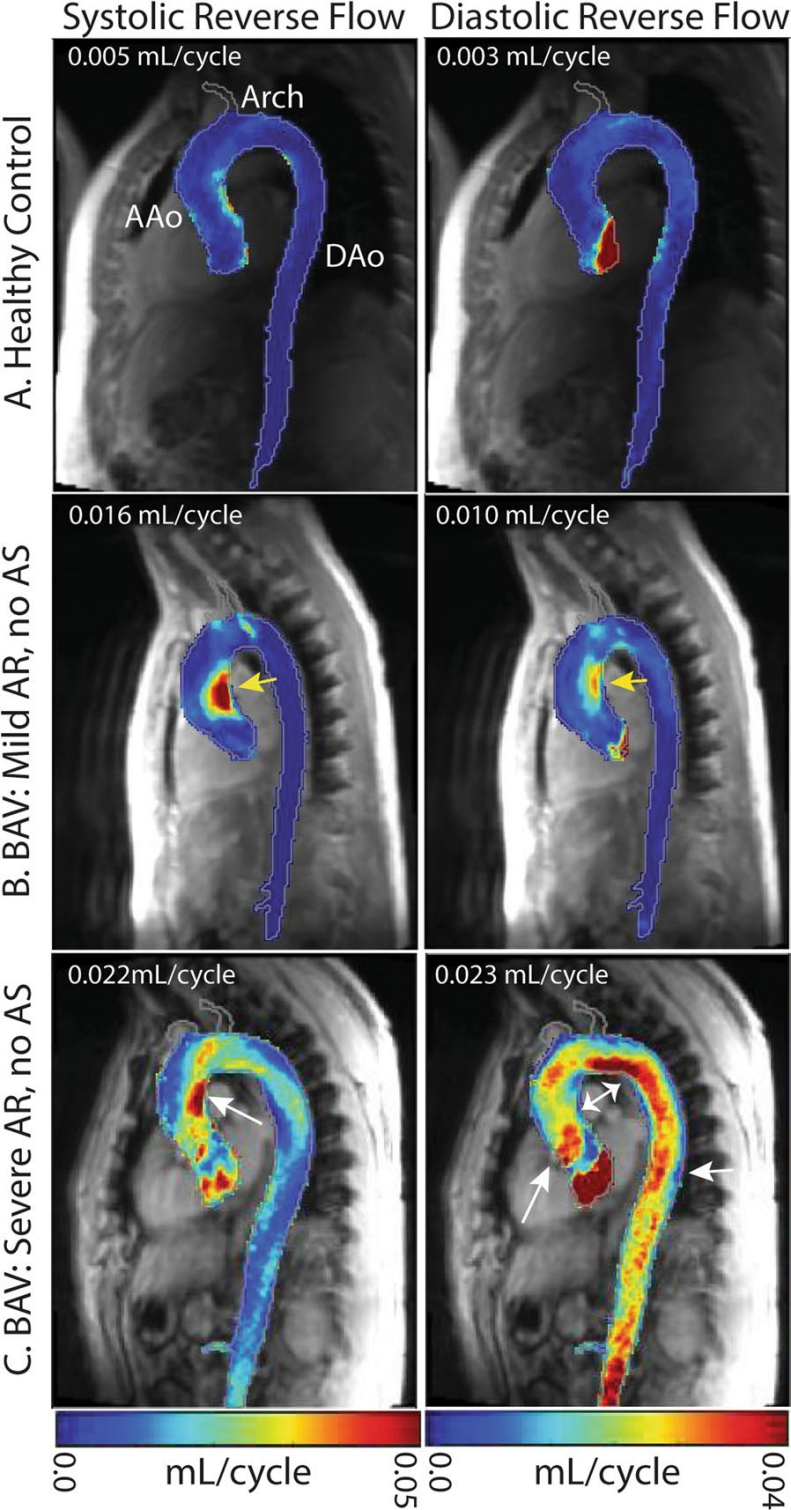
Reverse flow mean intensity projections of the aorta in a healthy control and BAV patients with isolated AR. Voxel-wise reverse flow in the AAo is reported in the upper left corner of each image. Systolic and diastolic reverse flow were increased in a subject with mild AR in the AAo (**b**, yellow arrows) compared to a healthy control (**a**). Diastolic flow increased along the entire aorta in the subject with severe AR (**c**) while systolic reverse flow remained similar and localized to the AAo (white arrows)

Figure 3 shows representative examples for BAV patients with isolated mild and severe AR but no AS. As expected, the presence of mild AR resulted in more pronounced systolic and diastolic reverse flow compared to a healthy control subject. For the BAV patient with severe isolated AR (no AS), diastolic reverse flow markedly increased along the entire aorta (0.01 vs. 0.023 mL/cycle) while systolic reverse flow remained localized to the AAo and similar compared to the mild AR BAV patient. The findings depicted in Figs. 2 and 3 were corroborated by results across the entire study cohort as detailed below.

To provide clinical context for the small, voxel-wise reverse flow values, we estimated the total reverse flow through a plane using measured reverse systolic flow for the two BAV patients presented in Fig. 2. For the patient with mild AS and a mean systolic reverse flow of 0.019 mL per voxel (Fig. 2b), we estimated 27.3 mL of reverse flow. For the subject with severe AS and a mean systolic reverse flow of 0.029 mL per voxel (Fig. 2c), we estimated 41.7 mL of reverse flow.

Bland–Altman analysis found excellent agreement in measurement of systolic (bias: 1.0e-8 mL/cycle, p = 0.44; LOA: 0.0007 mL/cycle) and diastolic (bias: 4.6e–5 mL/cycle, p = 0.65; LOA: 0.001 mL/cycle) reverse flow in the AAo. The coefficient of variation was less than 8% for both metrics, and the Pearson correlation coefficient was > 0.99 for both metrics.

### Reverse flow in BAV patients without AS and AR vs. TAV dilation and healthy controls

**Fig. 4:**
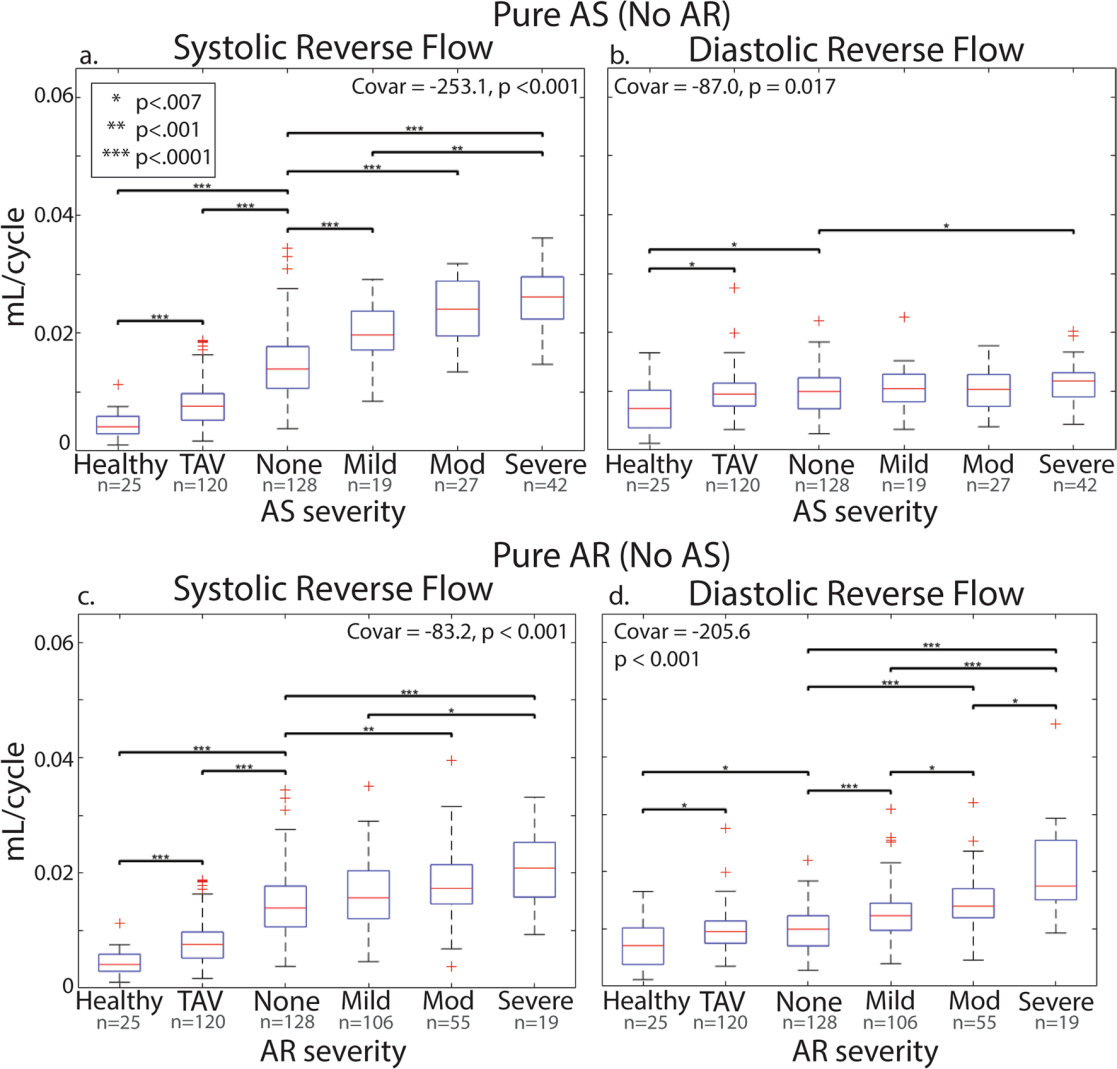
Reverse flow in BAV patients with isolated AS or AR. Mean systolic and diastolic reverse flow in the AAo as a function of AS severity (**a**, **b**) and AR severity (**c**, **d**). The covariate for logistic regression is reported with its p-value in each plot. Both AS and AR severity were significantly associated with increasing systolic and diastolic reverse flow. None = no aortic regurgitation or stenosis, Mod = moderate aortic regurgitation (AR) or aortic stenosis (AS), TAV = cohort with trileaflet aortic valve and aortic dilation

Whole cycle reverse flow in the ascending aorta in BAV patients without AR or AS was significantly increased compared to healthy controls (94% increase, p < 0.001). Similarly, systolic and diastolic reverse flow in the AAo were significantly elevated (222% increase, p < 0.001; 13.2% increase p < 0.01) compared to healthy controls without either AR or AS (Fig. 4). Systolic reverse flow was significantly increased in BAV subjects without AR or AS compared to TAV patients with dilation (79.3% increase, p < 0.0001, Fig. 4). There was no difference in diastolic reverse flow (p = 0.6). However, both systolic (79.2% increase, p < 0.0001) and diastolic (13.1% increase, p = 0.003) reverse flow were elevated in TAV patients with dilation compared to healthy controls (Fig. 4).

### Reverse flow in BAV patients with isolated AS or AR

To investigate the impact of isolated AS (Fig. 4a, b) or isolated AR (Fig. 4c, d) on AAo reverse flow, the cohort was divided into those without AS (n = 308) and those without AR (n = 216). In each subgroup, we compared systolic (Fig. 4a, c) and diastolic (Fig. 4b, d) reverse flow across AS and AR severity groups. In patients with isolated AR (no AS), diastolic AAo reverse flow increased with substantially AR severity (c = − 205.6, p < 0.001) and systolic AAo reverse flow increased moderately with AR severity (c = − 83.2, p < 0.001). In contrast, AS severity was strongly associated with an increase in systolic (c = − 253.1, p < 0.001) and moderately with diastolic (c = − 87, p < 0.001) AAo reverse flow.

### Interactions of AS or AR and impact on aortic reverse flow

**Fig. 5:**
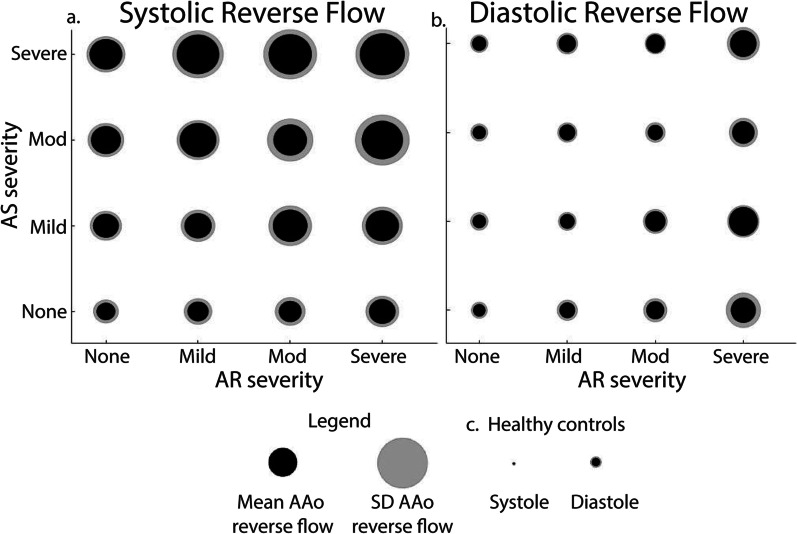
Mixed valve disease reverse flow. The BAV cohort was stratified by both AR and AS severity for systolic (**a**) and diastolic (**b**) reverse flow in the AAo. The mean reverse flow in the AAo (radius of the black circle) and the mean + 1 standard deviation (radius of the grey circle) are reported. The radii linearly scale with the mean and standard deviation. Systolic and diastolic reverse flow for healthy controls were visualized for reference (**c**). None = no aortic regurgitation or stenosis, Mod = moderate aortic regurgitation or stenosis

In Fig. 5, BAV patients were grouped by AR and AS status and arranged in an AS-AR severity matrix – allowing for mixed valve disease groups. Mean systolic (Fig. 5a) and diastolic (Fig. 5b) AAo reverse flow for each group are visualized by black circles (radius = mean AAo reverse flow). The variability of AAo reverse flow for each group is depicted by the outer grey circles (radius = SD of AAo reverse flow). Systolic ascending aorta reverse flow (Fig. 5a) increased with AS. Notably, for a given AS severity, AR severity had little impact on systolic AAo reverse flow. We found that only those without AS and those with severe AS showed a significant increase in systolic reverse flow when comparing those without AR to those with severe AR. (43.7% increase, p < 0.001; 38.4% increase, p < 0.001). Conversely, diastolic reverse flow (Fig. 5b) increased with AR severity. AS severity had significant impact on diastolic AAo reverse flow only in those without AR (16.5% p < 0.001). Thus, there was minimal interaction between AS and AR on ascending aorta systolic or diastolic reverse flow.

### Impact of bicuspid aortic valve morphology on reverse flow

**Fig. 6:**
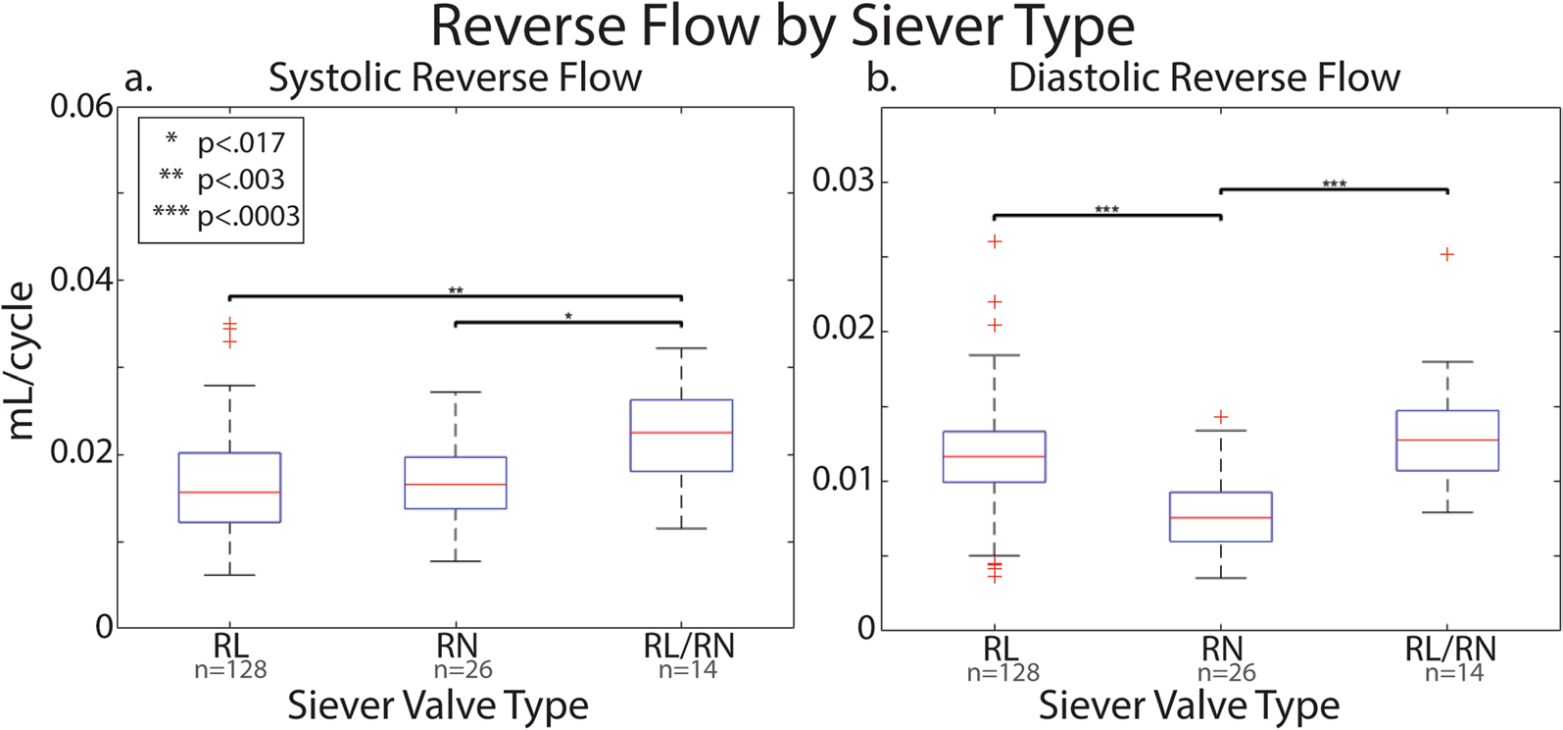
Ascending aortic systolic (**a**) and diastolic (**b**) reverse flow in BAV patients with no or mild AR and AS stratified by valve morphology

Among the BAV cohort, three valve morphologies had greater than 10 subjects: right and non-coronary leaflet fusion (RN, n = 26), right and left leaflet fusion (RL, n = 128) and RL and RN fusion (RL/RN, n = 14). Those with RL/RN had a significantly increased systolic reverse flow compared to patients with RN (0.020 vs. 0.017 mL, p = 0.006) and RL (0.020 vs. 0.016 mL, p = 0.001; Fig. 6a). Additionally, those with RN valves had significantly less diastolic flow in the AAo compared to those with RL (0.008 vs. 0.011 mL, p < 0.001) and RL/RN (0.008 vs. 0.014 mL, p < 0.001; Fig. 6b). There was no significant difference in distribution of no vs. mild AR across the three groups. RL/RN had a significantly greater proportion of subjects with mild AS than RN and RL (50% vs. 9% and 15% respectively, p < 0.01).

### Reverse flow and aortic diameter

Across the entire BAV cohort, increased systolic and diastolic reverse flow in the AAo were mildly but significantly associated with larger MAAD (r = 0.22, p < 0.0001, r = 0.09, p < 0.05). Multiple linear regression (Table 2) showed that ASs, AR, LVEF, SV, and gender were also significant independent predictors of systolic reverse flow. Notably, both AS and AR severity had significant contributions to the model. However, AS severity provided the strongest associations with increasing systolic AAo reverse flow. For the diastolic reverse flow model, neither MAAD nor AS severity were significant independent predictors of diastolic ascending aorta reverse flow.

**Table 2:** Multiple linear regression for systolic and diastolic reverse flow

Reverse flow	MAAD	AS			AR			LVEF	SV	Gender (male)	Age	R^2^	p-value
		Mild	Mod	Severe	Mild	Mod	Severe						
Systolic	3e−4***	0.005***	0.009***	0.01***	0.002***	0.003***	0.006***	1e−4**	5e−5***	− 0.002**	1e−5	0.54	1e−72
Diastolic	− 2e−5	7e−4	− 5−e	7e−4	0.002***	0.003***	0.008***	− 7e−5***	3e−5***	0.002***	5e−5^**^	0.39	1e−43

AR, AS, LVEF, SV, gender, age and MAA diameter were included in this model due to significant correlation with aortic reverse flow. The regression coefficients are reported, and coefficient significance is denoted by asterisk. MAAD was a significant contributor to systolic, but not diastolic, reverse flow in the ascending aorta (AAo). p-value legend: *p < 0.05, **p < 0.01, ***p < 0.001

## Discussion

Voxel-wise reverse flow mapping, a direct measure from 4D flow CMR, is an easy to obtain, volumetric measure that is associated with the presence and severity of aortic valve disease and aortic dilation in patients with BAV. Our main findings were (1) both systolic and diastolic AAo reverse flow were elevated in BAV patients compared to healthy controls and systolic reverse flow was elevated compared to TAV patients with dilation even in the absence of AS and AR; (2) systolic ascending aorta reverse flow increased with both AR and AS severity, (3) AR severity was the main driver of diastolic AAo reverse flow; (4) there was little evidence that AS and AR interact with respect to increasing systolic and diastolic AAo reverse flow; and (5) increased systolic AAo reverse flow was independently associated with MAAD.

BAV, even in the absence of AS or AR, is known to cause systolic flow jets as well as other deranged flow patterns [16, 31–33]. Specifically, BAV can drive the emergence of vortex and helical flow during systole that persists into diastole and throughout most of the cardiac cycle. These patterns, in addition to the flow jets, tend to localize to the AAo [16, 32–34]. Voxel-wise reverse flow maps captured this localized BAV mediated flow disturbance in systole and diastole, even in the absence of AS and AR. This is congruence with a prior study [23], finding increased reverse flow over the whole cardiac cycle in a heterogenous cohort of BAV patients.

AS has been shown to exacerbate BAV related systolic jets, [19, 31, 34, 35] which can further drive vortex flow in the AAo. We were able to quantitatively capture this increase in abnormal flow with reverse flow maps. We found a significant increase in systolic reverse flow in the AAo, likely reflecting increased jetting and vortex flow, and a significant, though less substantial, increase in diastolic reverse flow, likely reflecting additional persistent vortex flow.

AR is characterized by regurgitant flow through the aortic valve and has a distinctly different mechanism than AS for driving deranged flow. Rather than increasing flow velocities during ejection, AR causes regurgitant flow towards the leaky valve and is considered a diastolic phenomenon. Aortic reverse flow maps captured this regurgitant flow, as we found diastolic reverse flow in the AAo increased significantly with AR severity. We also found that systolic reverse flow in the AAo associated with AR severity, though to a lesser extent. This likely reflects flow reversal in the ascending aorta prior to AR occurring at the level of the aortic valve in early diastole.

Given the distinct mechanisms for AS and AR, their interaction effect on reverse flow in the Ao has previously not been fully explored and understood. The findings in our study cohort provide new evidence that there is minimal interaction between AS and AR. AS severity was the main driver of systolic reverse flow in the AAo, largely independent of AR severity. Similarly, AR severity was the predominant cause for increased diastolic AAo reverse flow regardless of AS severity. These observations suggests that 4D flow derived voxel-wise reverse flow maps can be a useful tool to isolate the impact of comorbid AS and AR on distinct changes in 3D blood flow dynamics in the aorta.

In a subset of the BAV cohort, we investigated the impact of valve morphology on reverse flow. We found that those with RN/RL had increased systolic reverse flow. This is in line with previous studies that found RN/RL morphology was associated with increase systolic WSS [36] and angle of flow jet [37]. Those with RN type valves had less diastolic reverse flow. The impact of valve type on diastolic flow has not been well studied. However, it has been shown that RN morphology tends to drive increased WSS and flow displacement in the distal ascending aorta compared to RL [35]. This localization may decrease the amount of retrograde flow. Of note, the RN/RL group had more patients with mild AS and a larger cohort analysis would be needed to investigate if the differences in systolic reverse flow were due to stenosis severity or valve morphology.

We found an independent association between increased AAo reverse flow and larger MAAD. This relationship may be due to an increased aortic diameter creating additional space for vortex flow to develop adjacent to the systolic outflow jets. We found this diameter-flow relationship for systolic, but not diastolic, reverse flow in the AAo, suggesting that systolic reverse flow in the AAo is the most sensitive parameter for detecting deranged flow in BAV patients with aortic dilation. While other studies [38–40] have found an association between systolic reverse flow and aortic diameter, these studies were severely limited by their lack of inclusion of subjects with AS and mixed valve disease. These studies also did not make use of the complete volumetric data, analyzing flow only at 2D planes, and were unable to produce flow maps to visualize reverse flow over the entire aorta.

We found excellent interobserver agreement for both systolic and diastolic reverse flow in the AAo. This suggests that the semi-automated analysis pipeline to measure systolic and diastolic reverse flow, is highly repeatable, similar to findings in a prior study [29]. Additionally, Kilinc et al. [41] demonstrated scan-rescan repeatability of voxel-wise reverse flow in a cohort of 12 type B aortic dissection patients and 2 healthy subjects. They report inter-class correlation coefficients > 0.9 across three 4D flow CMR acquisitions in the true lumen, indicating excellent reproducibility. Despite differences in the cohort composition and regions of interest, the reported mean reverse flow in the true lumen (0.016 mL/cycle) is within the range of values we measured in this study. Thus, both 4D flow derived voxel-wise reverse flow and the analysis pipeline show high degrees of reproducibility.

### Limitations

Our study has several limitations. Our study used a retrospective patient enrollment without longitudinal follow-up data. We were unable to assess the predictive value of aortic reverse flow for future complications such as accelerated aortic growth, need for surgical intervention, or other adverse outcomes. A large follow-up cohort would be ideal to discern if reverse flow can predict aortic growth or adverse outcomes. We were also limited by a small healthy control cohort which may limit the statistical significance of the observe differences in reverse flow.

Another limitation of our study is that we did not evaluate the impact of 4D flow CMR spatial resolution or slab thickness on reverse flow mapping. While all data was interpolated to 1 mm isotropic resolution, it is possible that varied acquired voxel-sizes would impact reverse flow measurements. It is possible that smaller voxels, with inherently reduced signal-to-noise ratio, may produce noisier measurements whereas larger voxels may capture neighboring regions of forward and reverse flow which could reduce the measured reverse flow. Future studies systematically comparing scans with different acquired resolutions, but in patients with identical valve disease status, should be performed to assess this limitation.

The cohort had a heterogonous mix of scanning parameters and protocols. We did not systematically investigate the impact of B_o_ strength, aortic wall motion, venc, or truncation of diastole to ensure equal scan duration. With respect to B_o_ strength, our cohort included < 5 subjects at 3T and we expect the impact of any differences to be minimal. Another limitation is the use of a static aortic mask. It has been shown in other metrics, that aortic wall motion can significantly impact hemodynamic metrics, such as WSS. Since reverse flow is a voxel-wise luminal measurement over a large 3D aortic region, we do not expect a substantial impact. Additionally, there was a large range of vencs used. However, only cases with appropriate antialiasing were included. We also expect the difference in venc to manifest during diastole, where velocities are lower, and that there should be little effect on systolic measurements. Our method excludes late diastole, which minimizes the impact of venc differences, but also means that diastolic reverse flow was not completely captured. It is possible there are BAV or dilation related hemodynamic changes occurring in late diastole that were not detected by our method. Further studies with retrospectively electrocardiogram gated 4D flow CMR sequences are needed to investigate the role of late diastolic flow.

A potential drawback of our method is the use of flow in the entire aorta to determine end systole as opposed to using the ascending aorta or valve plane. The use of the AAo segments was first tested and compared with the timing of end-systole obtained from with mid-ventricular short-axis CINE data. It was found that this method would select erroneous time points for end-systole in some cases, potentially due to substantial systolic reverse flow in this region. When using the whole aorta, end systole was correctly identified even in cases where the ascending aorta method failed.

## Conclusion

4D flow CMR derived reverse flow in systole and diastole is an easy to obtain metric that is associated with the presence of BAV, severity of aortic valve disease, and aortic dilation. The metric successfully captured BAV aortopathy related reverse flow in both systole and diastole compared to healthy controls. We demonstrated that systolic reverse flow was elevated in BAV patients compared to TAV patients with aortic dilation and that both AS and AR severity contributed to the extent of systolic reverse flow. This work suggests that systolic reverse flow, which increased with AS severity and MAA diameter, should be investigated as a potential metric for improving risk stratification of BAV patients with aortic dilation in future longitudinal studies.

## Data Availability

The data that support the findings of this study are available on request from the corresponding author EKW. The data are not publicly available due to data sets containing information that could compromise research participant privacy/consent.

## Abbreviations

AAo: Ascending aorta
AR: Aortic regurgitation
AS: Aortic stenosis
BAV: Bicuspid aortic valve
BMI: Body mass index
CMR: Cardiovascular magnetic resonance
CO: Cardiac output
DAo: Descending aorta
LV: Left ventricle/left ventricular
LVEDV: Left ventricular end-diastolic volume
LVEF: Left ventricular ejection fraction
LVESV: Left ventricular end-systolic volume
MAAD: Mid-ascending aorta diameter
SV: Stroke volume
TAV: Trileaflet aortic valve
WSS: Wall shear stress
